# A new standard radiographic reference for proximal fibular height in children

**DOI:** 10.1080/17453674.2020.1769378

**Published:** 2020-05-26

**Authors:** Adrien Frommer, Maike Niemann, Georg Gosheger, Gregor Toporowski, Andrea Laufer, Maria Eveslage, Jan Niklas Bröking, Robert Rödl, Bjoern Vogt

**Affiliations:** a Children’s Orthopedics, Deformity Reconstruction and Foot Surgery, University Hospital of Muenster;; b General Orthopedics and Tumor Orthopedics, University Hospital of Muenster;; c Institute of Biostatistics and Clinical Research, University of Muenster, Germany

## Abstract

Background and purpose — To date there is a lack of studies defining the anatomical position of the proximal fibula. This is especially relevant when planning surgical interventions affecting the knee joint such as permanent or temporary epiphysiodesis to correct leg length discrepancies or angular deformities in growing patients. The goal of this study is to establish a standardized measurement technique and radiological reference values for the position of the proximal fibula in children.

Patients and methods — 500 measurements were performed in calibrated long standing anteroposterior radiographs of 256 skeletally immature patients (8–16 years; 233 female, 267 male legs). As a radiographic reference in the frontal plane, the distance between the center of the proximal tibial growth plate and a line tangential to the tip of the fibular head and horizontal to the imaging plane was measured (dPTFH).

Results — The average value of dPTFH in the studied population (median age 12 years) was –2.7 mm (SD 3, CI –3.0 to –2.5) and normally distributed (p = 0.1). There were no clinically significant sex or age-dependent differences. The inter-rater reliability analysis showed excellent ICC values (ICC = 0.88; CI 0.77–0.93).

Interpretation — This study provides a new radiographic reference value to assess the position of the proximal fibula in relation to the proximal tibia in children and adolescents. This reference can aid preoperative decision-making as to whether additional fibular epiphysiodesis is necessary when performing tibial epiphysiodesis to correct moderate leg-length discrepancies.

Detailed knowledge of the physiological limb alignment is of importance for the treatment of limb deformities (Moreland et al. 1987, Chao et al. 1994, Paley 2002). Joint orientation angles of the lower limb and numerical reference values such as the mechanical axis deviation (MAD) help to differentiate physiological from pathological limb alignment (Moreland et al. 1987, Paley 2002) when planning procedures such as corrective osteotomies or epiphysiodesis (ED) for correction of angular deformities or moderate leg-length discrepancies (LLD) (Bowen and Johnson 1984, Canale and Christian 1990, Vogt et al. 2014). While these references aid the assessment of knee joint alignment, to date there is a lack of standardized radiographic references to evaluate the anatomical location of the fibular head in relation to the proximal tibia. The position of the proximal fibula is clinically relevant when planning temporary or permanent tibial ED for moderate LLD. When planning tibial ED the knowledge of remaining growth potential and the presence of pre-existing fibular overgrowth is essential (McCarthy et al. 2003). Some surgeons favor performing a concomitant fibular ED with tibial ED to prevent fibular overgrowth, which might cause discomfort and instability of the knee joint due to laxity of the lateral collateral ligament (LCL) (Canale and Christian 1990, Porat et al. 1991, Metaizeau et al. 1998, McCarthy et al. 2003). Others argue that fibular ED should not be performed due to the risk of peroneal nerve injury and claim the amount of overgrowth is irrelevant (Bowen and Johnson 1984, Gabriel et al. 1994, Siedhoff et al. 2014). Different radiographic approaches have been described previously to measure proximal fibular overgrowth or shortening (Ogilvie and King 1990, McCarthy et al. 2003, Kim et al. 2019) but until today there is no standardized radiographic reference defining the anatomical location of the proximal fibula in children aged 8–16 years.

The goal of this study is to define a new radiographic reference for the position of the proximal fibula in skeletally immature patients and to test whether there are age- and sex-dependent differences. We believe that the results can help clinical decision-making, especially in the treatment of LLD by tibial ED in children.

## Patients and methods

The studied radiographs were all obtained from the archives of our orthopedic clinic from the past 10 years. Most of the radiographs originate from patients who were treated in the outpatient department of our institution. The most common indications for the radiographic examination were: ruling out pathological limb alignment, follow-up of permanent or temporary isolated femoral ED for LLD or angular deformities, and assessment of LLD.

Calibrated long-standing anteroposterior radiographs were retrospectively analyzed from a population of 256 skeletally immature patients with a chronological age of 8 to 16 years. This period of age typically represents the time when growth-dependent surgical procedures of the lower limb can be performed. The radiographs were evaluated retrospectively with the following inclusion criteria: chronological patient age at radiologic examination 8–16 years, LLD < 1 cm, MAD < ± 2 cm. Radiographs from patients who underwent operative treatment of the knee joint, who received systemic treatment like chemotherapy or growth hormone application, and who had evidence of maltorsion, congenital disorders, or history of trauma of the leg were excluded from the study. If both legs of one patient met our inclusion criteria bilateral measurements were conducted. In unilateral congenital disorders or LLD the unaffected contralateral leg was included in the study. This resulted in a radiological assessment of 500 legs, 233 female (f) and 267 male (m) legs.

Long standing radiographs were obtained by digitally stitching 3–4 (depending on the individual’s leg length) sector radiographs together. The images were captured from a defined distance (2.8 m) with a metal calibration sphere (25.4 mm diameter) mounted on an adjustable flexible arm.

**Figure 1.**
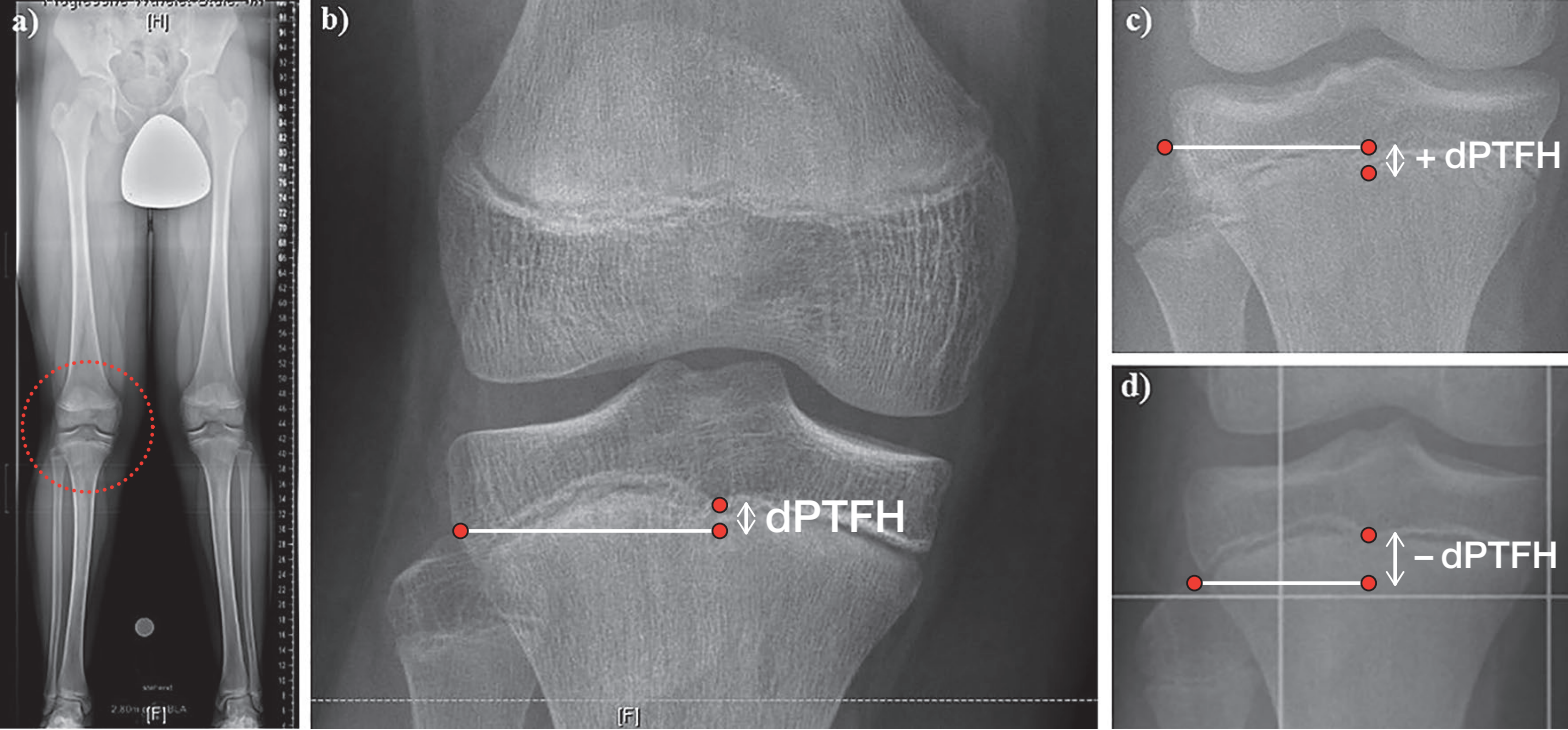
(a) The distance between the center of the proximal tibial growth plate and the tip of the fibular head (dPTFH) is measured in the frontal plane of long standing radiographs of a 13-year-old boy. (b) dPTFH is defined by the distance in millimeters between the center of the tibial growth plate and a line tangential to the tip of the fibular head and horizontal to the imaging plane. (c, d) Negative values indicate that the fibular head is localized more distally than the center of the proximal tibial growth plate and vice versa.

**Figure 2.**
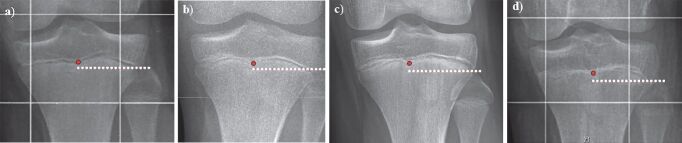
Radiological assessment of the center of the proximal tibial growth plate and the tip of the proximal fibula in order to measure dPTFH in different aged patients (a = 10 years, b = 12 years, c = 14 years, d = 16 years). While closer to skeletal maturity the growth plate appears less distinct (d), in general its outlines can still be estimated.

As a radiographic reference in the frontal plane, the distance between the center of the proximal tibial growth plate and a line tangential to the tip of the fibular head and horizontal to the imaging plane was measured in a way similar to previous studies (McCarthy et al. 2003) (Figure 1). Negative values indicate that the fibular head is localized more distally than the center of the proximal tibial growth plate and vice versa. In order to establish a standardized nomenclature, this value will be referred to as the “distance between the proximal tibial physis and the fibular head” (dPTFH). dPTFH was measured in the following age groups (AG): 8–10 years (n = 95: f/m = 44/51), 11–12 years (150: 75/75), 13–14 years (166: 75/91), 15–16 years (89: 39/50) (Figure 2).

3 observers (GT, NB, BV) independently measured dPTFH in 36 randomly chosen radiographs from the study population to assess the inter-rater reliability.

All measurements were performed on calibrated radiographs with the PACS® System (GE Healthcare, Chicago, IL, USA).

### Statistics

The statistical analyses were performed using IBM SPSS® Statistics 25 for Windows (IBM Corp, Armonk, NY, USA) and R version 3.6.1 (R Foundation for Statistical Computing, Vienna, Austria). Normal distribution of the measurements was assessed descriptively using a histogram. Data are reported as mean ± standard deviation (SD) and 95% confidence intervals (CI) for the mean. In order to account for intra-individual correlation, the effect of age and sex on the dPTFH values was analyzed using a linear mixed model (LMM). The model included age (centered at the mean), sex, and their interaction as fixed effects and a random intercept for the patient. The model fit was assessed descriptively using Q–Q plots. An additional mixed model was computed using the age groups as single fixed effect.

The intraclass correlation coefficient (ICC) for the 3 raters was estimated based on the estimated variance components of a linear mixed model including a random effect for each of the patient and the leg of the patient and a fixed effect for the rater. A 95% confidence interval was computed using a parametric bootstrap (10,000 runs). Sample size calculation for 3 independent raters was performed with PASS 16.0.4 (NCSS, LLC. Kaysville, UT, USA) by assessing the width of a 2-sided 95% CI for the ICC.

No adjustment for multiplicity was applied. All inferential statistics are intended to be exploratory, not confirmatory.

### Ethics, funding, and potential conflicts of interest

The study was approved by the ethical committee of the university of Muenster on November 21, 2017 (registration number: 2017-491-f-S). The authors received no funding for this work and have no conflict of interest.

## Results

**Figure 3.**
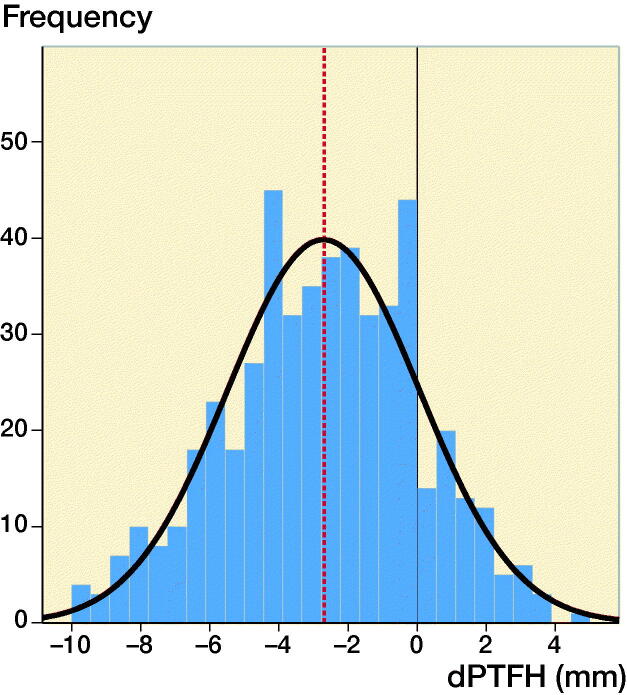
The graph demonstrates a normal distribution of dPTFH measured in 500 legs of children and adolescents from the age of 8–16 years. The mean dPTFH is –2.7 mm with a standard deviation (SD) of 2.8 mm. For clinical practicability mean and SD should be approximated to –3 mm and 3 mm, respectively.

**Figure 4.**
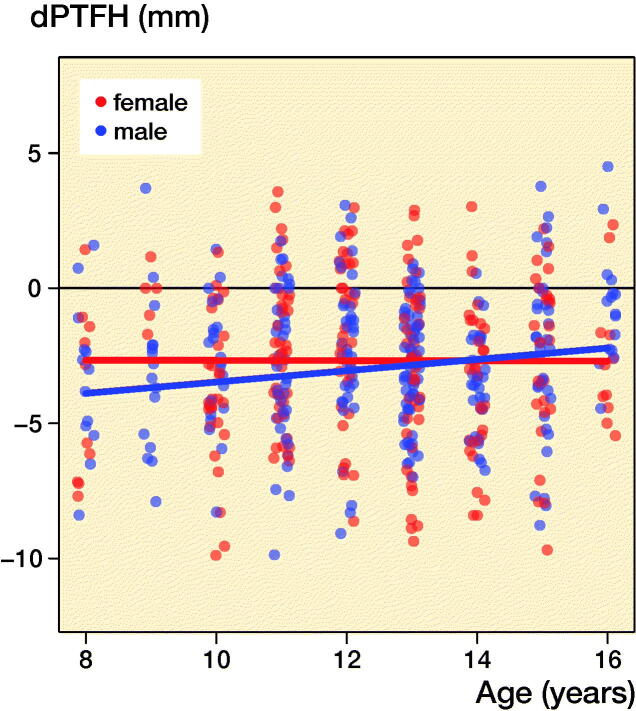
Scatterplot of age vs. dPTFH including the regression lines resulting from the linear mixed model presented in Table 2.

The median age was 12 years (8–16 years, mean 12.4 years) and the dPTFH was normally distributed. The average value of dPTFH was -2.7 mm (SD 2.8, CI -3.0 to -2.5) (Figure 3). The LMM analysis showed no statistically significant association with age or sex and no noticeable interaction between these 2 parameters (Figure 4, Tables 1 and 2). The age and sex dependent distribution of dPTFH revealed no clinically relevant difference (Figure 4). The following dPTFH values were measured in the defined AG: 8–10 years: dPTFH = –3.2 mm (CI –3.7 to –2.6), AG 11–12 years: dPTFH = –2.3 mm (CI –2.8 to –1.8), AG 13–14 years: dPTFH = –3.1 mm (CI –3.5 to –2.7), AG 15–16 years: dPTFH = –2.1 mm (CI –2.7 to –1.5) (Table 3). No statistically significant differences between the age groups were found in the LMM analysis (Table 2).

**Table 1.** Results of the linear mixed model for dPTFH (mm) including fixed effects for sex (centered at the mean), sex, and the interaction between age and sex

Factor	Regression coefficient (95% CI)	p-value
Intercept	–2.68 (–3.18 to –2.19)	< 0.001
Age (years)	–0.0049 (–0.23 to 0.22)	0.1
Sex (male vs. female)	–0.29 (–0.94 to 0.35)	0.4
Age x sex	0.21 (–0.12 to 0.55)	0.2

**Table 4.** Conditions with preexisting proximal fibular under- and overgrowth

Fibular undergrowth	Fibular overgrowth
Idiopathic	Idiopathic
Posttraumatic or infectious	Posttraumatic or infectious
(e.g., damage to the fibular	(e.g., damage to the tibial
growth plate)	growth plate)
Congenital	Congenital
(e.g., femoral deficiency,	(e.g., achondroplasia,
fibular hemimelia, etc.)	Desbuquois dysplasia, etc.)

**Table 3.** Mean dPTFH in the age groups

Age group	Sample (n)	Mean dPTFH (95% CI)(mm)
8–10	94	–3.2 (–3.7 to –2.6)
11–12	150	–2.3 (–2.8 to –1.8)
13–14	166	–3.1 (–3.5 to –2.7)
15–16	89	–2.1 (–2.7 to –1.5)

**Table 2.** Results of the linear mixed model for dPTFH (mm) including age group as a fixed effect

Factor	Regression coefficient (95% CI)	p–value
Intercept	–3.18 (–3.96 to –2.39)	< 0.001
Age group		
11–12 vs. 8–10	0.61 (–0.36 to 1.58)	0.2
13–14 vs. 8–10	0.081 (–0.83 to 0.99)	0.9
15–16 vs. 8–10	0.81 (–0.38 to 1.99)	0.2

The analysis showed no statistically relevant difference between the age groups.

For the ease of clinical practicability, the lack of clinical relevance, and taking measurement inaccuracy into consideration the values for the mean and SD of dPTFH can be approximated to –3 mm and 3 mm, respectively.

The estimated ICC value (ICC = 0.88; CI 0.77–0.93) showed excellent reliability for the measurements performed by 3 independent observers.

## Discussion

Standard radiographic references of joint and limb alignment are of fundamental importance for the treatment of limb deformities and leg-length discrepancies. Various studies have improved the field of deformity reconstruction by providing radiological reference values to distinguish between physiological and pathological limb alignment (Moreland et al. 1987, Chao et al. 1994, Paley 2002). When considering the lower leg, previous studies have mainly assessed the radiological location of the distal fibula in relation to the ankle joint. Ogden and McCarthy (1983) have shown that during adolescence the distal fibular physis is normally level with the tibial articular surface of the ankle joint. The Shenton line and dime sign are radiographic measurements that have been described in order to analyze the relationship of the distal fibula and distal tibia (Panchbhavi et al. 2018).

These reference values are commonly used in orthopedic and traumatological daily routine and help to analyze malleolar and ankle fractures and to radiologically control the results of surgical reduction (Ogden and McCarthy 1983, Weber and Simpson 1985, Panchbhavi et al. 2018).

However, to date there is a lack of standard radiographic reference values defining the anatomical position of the proximal fibula in relation to the proximal tibia in children and adolescents. Previous radiological examinations evaluated the proximal and distal “tibial–fibular physis distance” in 63 children from the age of 1 to 12 years (Beals and Skyhar 1984). These observations help the assessment of the tibio–fibular relation from an early age onward but do not provide standard radiographic reference values for adolescents in which growth-influencing surgeries are commonly performed.

Our findings should be seen in the context of different treatment options, especially for moderate LLD in skeletally immature patients. LLD of 2–5 cm can be an impairing condition affecting gait pattern and mobility. While at skeletal maturity lengthening procedures are commonly performed (Schiedel et al. 2014, Reitenbach et al. 2016, Horn et al. 2019), during childhood and adolescence temporary or permanent proximal tibial and/or distal femoral ED of the relatively longer leg are established methods to treat LLD (Canale and Christian 1990, Gabriel et al. 1994, Metaizeau et al. 1998, McCarthy et al. 2003, Siedhoff et al. 2014, Vogt et al. 2014, Boyle et al. 2017).

There is controversy in the literature as to whether an additional ED of the fibular head should be performed together with tibial ED. While some authors argue that fibular ED should be performed to prevent fibular overgrowth in relation to the arrested tibia and consequently laxity of the LCL (Canale and Christian 1990, Draganich et al. 1991, Metaizeau et al. 1998, McCarthy et al. 2003, LaPrade et al. 2010, Arikan and Misir 2019), others argue that the risk of peroneal nerve injury does not justify the intervention and that the possible amount of relative fibular overlength is clinically irrelevant (Bowen and Johnson 1984, Gabriel et al. 1994, Siedhoff et al. 2014).

Part of this controversy results from the lack of standard radiographic reference values to assess the anatomical height of the fibular head in relation to the proximal tibia. When planning the correction of moderate LLD by tibial ED the surgeon must consider if concomitant proximal fibular ED is necessary or not. Thus, it is essential to evaluate the localization of the proximal fibula in relation to the proximal tibia before the beginning of treatment.

The goal of the latter considerations is to maintain the physiological proximal tibio–fibular relation by prevention of secondary fibular overgrowth. On the other hand, a standard radiographic reference value might be of at least equal importance in conditions with pre-existing proximal fibular under- or overgrowth in relation to the tibia (Table 4). Especially in patients with significant LCL instability and subsequent gapping of the medial knee joint due to preexisting proximal fibular overgrowth, as can frequently be seen in achondroplasia, surgical correction of the tibio–fibular disproportion (e.g., fibular ED) can be considered (Lee et al. 2007).

**Figure 5.**
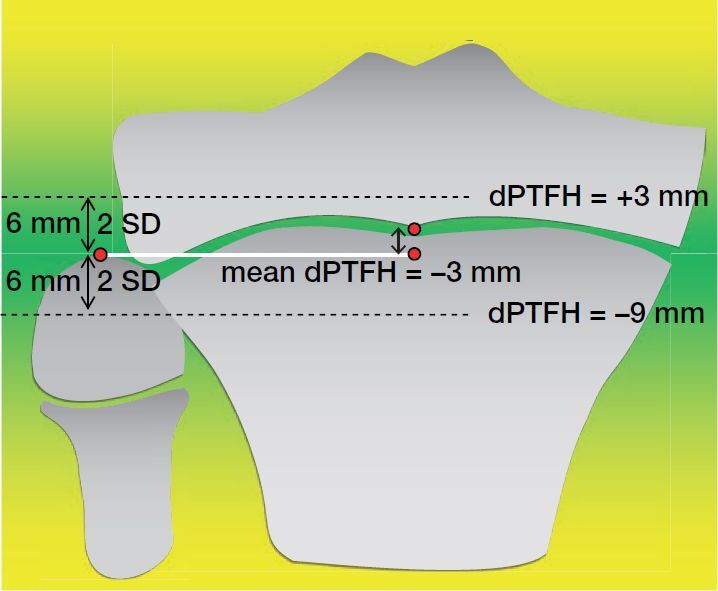
This study provides dPTFH as a new standard radiographic reference defining the anatomical localization of the fibular head in relation to the center of the proximal tibial growth plate. The mean dPTFH in children and adolescents (8–16 years) is –3 mm with an SD of 3 mm. We propose to consider deviations of dPTFH greater than 2 SD as fibular overlength (dPTFH > +3 mm) or shortening (dPTFH < –9 mm) respectively.

This study shows that the mean dPTFH in children and adolescents (8–16 years) is –3 mm with an SD of 3 mm. We propose to consider deviations of dPTFH greater than 2 SD as fibular overlength (dPTFH = +3 mm) or shortening (dPTFH = –9 mm), respectively (Figure 5).

The inter-rater reliability analysis has shown that dPTFH can be measured accurately and is reproducible by independent observers. These results indicate that dPTFH is a reliable measurement value that can be implemented in routine radiological limb alignment analysis.

Our results should be understood taking into consideration the following limitations. Different techniques of radiographic analysis can lead to variations in the dPTFH depending on the angle of the X-ray beam, therefore this study provides reference values for the height of the proximal fibula only in calibrated long standing, full-weight-bearing anteroposterior radiographs. This study does not supply clinical information regarding the stability of the knee joint or potential discomfort caused by proximal fibular overgrowth or shortening. Further studies will be needed to assess how a dPTFH greater than 2 SD affects the function of the knee joint.

As a new standard radiographic reference dPTFH can aid preoperative decision-making as to whether additional fibular ED is needed when performing tibial ED to correct moderate LLD in children and adolescents by defining the anatomical height of the proximal fibula.

AF: wrote the manuscript, performed and supervised the measurements of dPTFH, supervised the inter-rater reliability analysis, performed statistical analysis, and prepared the figures. MN: performed dPTFH measurements and statistical analysis. GG: provided the radiographs and made substantial changes to the manuscript. GT: performed the measurement for the inter-rater reliability analysis, and critically assessed and corrected the manuscript. AL: assessed and corrected the manuscript, arranged the data, and prepared the tables. ME: revised, performed, and wrote the statistical report. NB: performed the measurement for the inter-rater reliability analysis, and critically assessed and corrected the manuscript. RR: provided the radiographs, analyzed the data, supervised the work, and made substantial changes to the manuscript. BV: designed the study, analyzed the data, supervised the work, performed the measurement for the inter-rater reliability analysis, and critically assessed and corrected the manuscript.

The authors acknowledge support from the Open Access Publication Fund of the University of Münster, Germany.
